# Histoculture drug response assay predicts chemotherapy efficacy and improves survival in gastrointestinal cancers

**DOI:** 10.3389/fonc.2025.1596253

**Published:** 2025-07-16

**Authors:** Shuang Wang, Na Fan, Han Li, Yang Li, Ping Hu, Dongliang Chen, Xiaohong Jiang, Lei Gao, Chenggang Yang, Dawei Yang

**Affiliations:** ^1^ Zhong Yuan Academy of Biological Medicine, Liaocheng People’s Hospital, Liaocheng, China; ^2^ Deparment of Gastrointestinal Surgery, Liaocheng People’s Hospital, Liaocheng, China

**Keywords:** HDRA, gastrointestinal cancers, chemosensitivity, efficacy, prognosis

## Abstract

**Background:**

The high incidence, substantial mortality, and marked heterogeneity in chemotherapy responses among gastrointestinal tumors accentuate the imperative for individualized treatment strategies. This study aims to evaluate the reliability and clinical significance of the histoculture drug response assay (HDRA) in predicting chemotherapy sensitivity and prognosis. Specifically, it focuses on Chinese patients diagnosed with gastrointestinal cancers.

**Methods:**

This study enrolled 283 patients with gastrointestinal tumors, comprising 124 esophageal cancer cases, 92 gastric/cardia cancer cases, and 67 colorectal cancer cases. Immunohistochemistry was conducted to assess tumor structure integrity and the expression of Ki - 67, CD31, and E - cadherin before and after the HDRA assay. HDRA evaluated the efficacy and inhibition rates of single and combination chemotherapy regimens. Moreover, the effect of HDRA - guided treatment on patient survival was analyzed.

**Results:**

The results indicated that HDRA effectively preserved the three-dimensional structure and microenvironment of gastrointestinal tumors, as no significant changes were observed in the expression of Ki-67, CD31, or E-cadherin. Furthermore, combination regimens showed significantly higher efficacy and inhibition rates than single - agent therapies. Notably, platinum-based combination therapy was most effective in esophageal cancer. Survival analysis revealed that esophageal and gastric cancer patients receiving HDRA - sensitive regimens (HDRA group) had significantly longer disease - free survival (DFS) compared to those on non - sensitive regimens (N - HDRA group) and untreated patients. Cox regression analysis indicated that HDRA-guided treatment serves as a protective factor for DFS (hazard ratio, HR<1).

**Conclusion:**

In summary, the HDRA assay represents a reliable assay for accurately evaluating chemotherapy regimens, thereby furnishing guidance for individualized treatment in gastrointestinal cancer patients.

## Introduction

Gastrointestinal tumors, predominantly consisting of esophageal cancer, gastric cancer, colorectal cancer, are one of the most common cancers globally, marked by a high incidence and mortality rate (1, 2). Notably, due to the initial symptoms are not obvious, the majority of patients are diagnosed at the middle or advanced stages (3). In addition to surgical intervention, systemic chemotherapy or radiotherapy, and immunotherapy, either as monotherapies or in combination, are conventional clinical strategies aimed at improving patient prognosis (4, 5). For the majority of patients with gastrointestinal tumors, adjuvant chemotherapy remains the primary option for postoperative treatment (4, 6). However, due to the heterogeneity of tumors, individual patients may exhibit varying responses to chemotherapy. Clinical data indicate that the response rate of a specific chemotherapy regimen is only approximately 40% (7). In a global multi-center, randomized, double-blinded, Phase III clinical trial, among the Chinese subgroup population, the objective response rate (ORR) of the chemotherapy group for unresectable locally advanced or metastatic esophageal cancer was only 29.3% (8). In another Phase III clinical trial for patients who had previously untreated recurrent or metastatic gastric/gastroesophageal junction adenocarcinoma, the ORR of the chemotherapy group was 45% (9). Given that the efficacy of chemotherapy is associated with the patient’s sensitivity to the drug, there are inevitable individual differences in efficiency when choosing chemotherapy regimens if solely bases on guidelines or clinical experience. Improper treatment not only prolong the treatment period, but also leads to adverse effects (10). Consequently, the determination of the optimal individualized treatment strategy for each patient becomes a pivotal concern for clinicians.

The histoculture drug response assay (HDRA) with MTT endpoint has been used for testing chemosensitivity to address this significant clinical challenge (11). Compared to other drug sensitivity detection methods, the HDRA demonstrates notable advantages and clinical feasibility (12, 13). The 2D drug sensitivity test based on monolayer cell culture fails to accurately replicate the tumor microenvironment, poorly preserves tumor heterogeneity, and inaccurately simulates drug penetration, thereby not genuinely reflecting the clinical sensitivity characteristics of patients (14–18). Additionally, genomics focuses solely on the genetic level, overlooking the impact of the tumor microenvironment, metabolic heterogeneity (19, 20). Many variations are classified as ‘variants of uncertain significance’ (VUS), complicating direct medication guidance and incurring high costs (21). Compared to other drug sensitivity detection methods, the HDRA demonstrates notable advantages and clinical feasibility. The HDRA assay preserves the original structure of tissues and the natural microenvironment, maintaining tumor heterogeneity while accurately simulating the interaction between drug penetration and the microenvironment *in vivo (* 22). Although several studies have investigated the efficacy of HDRA with MTT endpoint in various cancers (23, 24), there are limited reports on the efficacy of chemotherapy drugs and the prognosis of gastrointestinal cancers. In particular, the application of HDRA in Chinese patients with gastrointestinal cancers has not been explored.

Since August 2020, we have conducted HDRA testing on patients with gastrointestinal cancers, including esophageal cancer, gastric/gastroesophageal junction adenocarcinoma cancer, colorectal cancer who have undergone operation in our hospital. The aim of this study is to elucidate the reliability and superiority of the HDRA assay in predicting the efficacy of chemotherapy and patient prognosis.

## Materials and methods

### Study population

This retrospective study included 283 patients with non-stage IV gastrointestinal tumors who underwent thoracic or gastrointestinal surgery and subsequently received HDRA testing at Liaocheng People’s Hospital between July 2020 and December 2023. The cohort comprised 124 patients with esophageal cancer, 92 patients with cardia/gastric cancer, and 67 patients with colorectal cancer. Based on the actual clinical treatments administered, patients were retrospectively categorized into three groups: the HDRA group, who received chemotherapy regimens containing drugs identified as sensitive by HDRA; the non-HDRA (N-HDRA) group, who, due to economic or clinical considerations, did not receive HDRA-guided therapy but were treated with alternative chemotherapy regimens; and the untreated group, who did not undergo chemotherapy owing to poor physical condition or personal choice. The follow-up time concluded at 31 August 2024, with a media follow-up duration of 22.87 months (ranging from 9.88 months to 35.86 months). All patients were informed in advance and signed consent forms. The present study was reviewed and approved by the Ethics Committee of Liaocheng People’s Hospital (No. 2019121).

### HDRA assay with MTT endpoint

Day 1: Fresh tumor specimens obtained through surgery were placed in a sterile preservation solution and subsequently transferred to the laboratory, where they were immediately washed with a cleaning solution. The cancer tissue sample was then cut into 60 pieces, each with a volume of approximately 0.5 to 1 cubic millimeter, and washed four times with the cleaning solution. These tissue pieces were subsequently placed in a culture medium, to which a tetrazolium compound solution was added, and then incubated at 37°C for 20 minutes in a CO_2_ incubator. The dark purple tissue pieces exhibiting high activity were selected and transferred to a new culture dish for overnight culture.

Day 2: The tissue samples were placed in a sterile 96-well plate, with two pieces of tissue per well, and subsequently cultured with either single or combined chemotherapy agents. Two duplicate wells were designated for each drug, while phosphate-buffered saline (PBS) served as the control.

Day5: The inhibition rate (IR) of each drug was assessed using the MTT assay. After removing 20 μL of culture medium, a succinate dehydrogenase complex solution was added, and culture for 4 hours. Following the removal of the supernatant, each well was treated with 150 μL of DMSO. The absorbance of the solution in each well was measured at 540 nm. The average absorbance from two parallel culture wells was used to calculate the absorbance for each drug agent.


$$\text{The inhibition rate }\left(\%\right)=\frac{1-\text{mean absorbance of treated tumor}}{\text{mean absorbance of control tumor}}\;\times \;100$$

An inhibition rate exceeding 30% indicates that the single or combination drugs demonstrate chemosensitivity. If the inhibition rate of the drug is negative, it is considered zero, indicating no chemosensitivity.

Anticancer drugs and plans: The anticancer drugs utilized for HDRA testing include TAX, DTAX, ALT, CDDP, LBP, CBP, OXA, TS-1, XEL,5-FU, CPT-11, and LEU. Among them, the testing drugs and plans for patients with esophageal cancer are TAX, ALT, CBP, LBP, as well as combination of TAX with CBP/LBP/CDDP, and ALT with CBP/LBP/CDDP. For patients with cardia/gastric cancer patients, the testing drugs and plans are TS-1, TAX, DTAX, and combinations of CBP with TAX, 5-FU with TAX/CDDP/DTAX, OXA with TS-1/5-FU and LEU/DTAX, 5-FU and LEU. In cases of colorectal cancer patients, the testing drugs and plans are 5-FU, XEL, OXA, CPT-11, TS-1, as well as combination of XEL with OXA/CPT-11, and 5-FU with LEU and OXA/CPT-11/OXA and CPT-11. The above required reagents and drugs are supplied by Antaikang Technology Co., LTD.

### Immunohistochemistry protocol

Specimen preparation: The surgically resected esophageal cancer tissue was cut into pieces measuring 0.5 cm × 0.5 cm × 0.5 cm prior to HDRA testing and following the HDRA process. These specimens were fixed in 4% neutral formalin for a duration of 24 to 48 hours, subsequently dehydrated in graded ethanol, cleared in xylene, and then immersed in wax at 65°C overnight. The samples were then embedded in wax blocks, sectioned to a thickness of 4-5 μm, and baked at 60°C.

Immunohistochemical staining: Paraffin sections were dewaxed with xylene, hydrated with graded ethanol, antigen retrieval with sodium citrate. Antigen retrieval was achieved with sodium citrate, followed by treatment with 3% hydrogen peroxide for 30 minutes to inhibit endogenous peroxidase activity. The samples were washed three times with phosphate-buffered saline (PBS) and then blocked with an immunohistochemistry blocking solution for 30 minutes. Primary antibodies, specifically Ki-67, CD31, and E-cadherin, were incubated overnight, and then washed the samples three times with PBS. The HRP-conjugated secondary antibody was incubated for one hour at room temperature and then washed three times with PBS. DAB chromogenic solution was added, and color development was typically observed for 3 minutes; the reaction was terminated by rinsing with distilled water once a clear brown positive signal was visible.

Results assessment: Two professional technicians observed the distribution and intensity of positive signals using a microscope. ImageJ software was employed to evaluate the expression levels of Ki-67, CD31, and E-Cadherin before and after the HDRA by the quantification of immunohistochemical staining using the following formula: Average Optical Density (AOD) =Integral optical density (IOD)/Area, and the higher AOD value indicates a higher level of protein expression.

### Statistical analyses

Data analysis was performed using SPSS ver.25.0. Continuous data were analyzed by the independent samples t-test or one-way ANOVA test. Tukey’s multiple comparison test was employed for between-group analysis. Categorical data were analyzed by χ2 test. When the data failed to meet the assumptions of normal distribution or homogeneity of variances, the Wilcoxon Rank-Sum Test and Kruskal-Wallis Test was applied for analysis. The bar chart was made by GraphPad Prism 8. Survival analysis and Cox regression model analysis were conducted using the R programming language. A p-value less than 0.05 was considered statistically significant.

## Results

### The feasibility of HDRA

**Figure 1:**
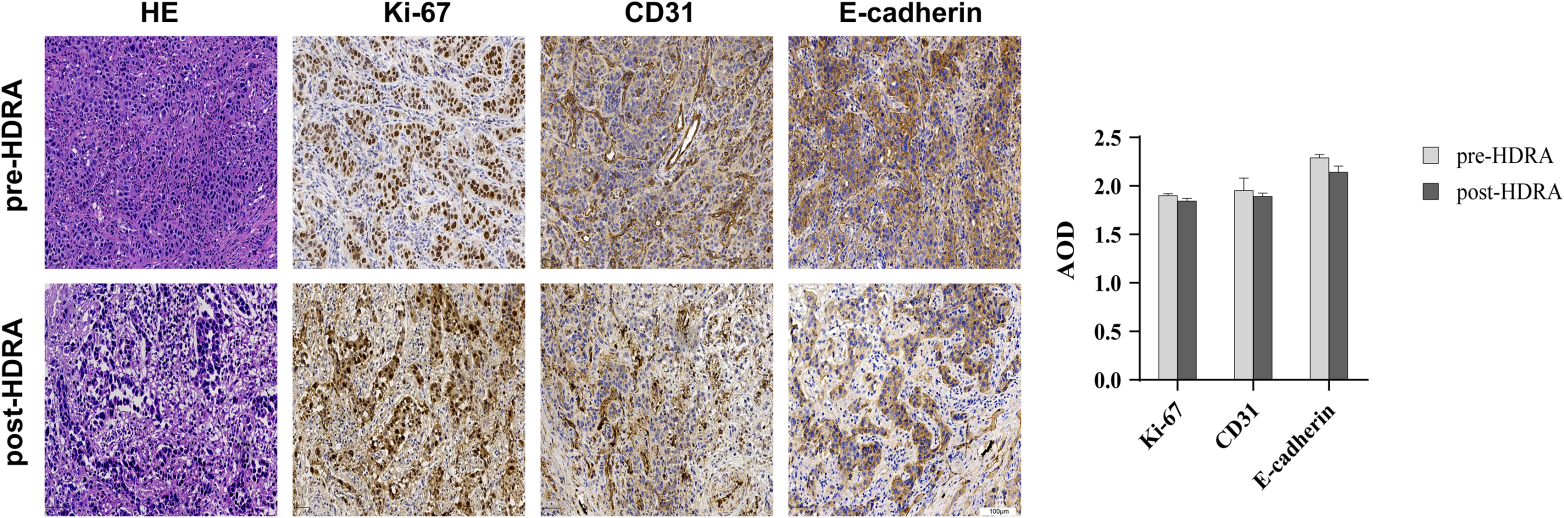
Comparison of esophageal cancer tissue structure and specific protein expression before and after HDRA. Tissues were stained with hematoxylin and eosin (H&E), and antibodies to the proliferation marker Ki67, vascular endothelial marker CD31, calcineurin-dependent adhesion molecule E-cadherin (20×magnification, bars=100um). Average Optical Density (AOD) of Ki-67, CD31, E-cadherin was performed by column chart.

To validate that the HDRA method in preserving the original characteristics of the patient’s tumor tissue and effectively guide personalized medication for precise treatment, HE staining and immunohistochemical staining were performed on esophageal cancer tissue to observe differences in tissue structure and specific protein expression before and after HDRA. Proteins of used included Ki-67, which represents tissue proliferation activity; CD31, which indicates vascular structure; and E-cadherin, which is associated with cancer cell metastasis (**Figure 1**). After undergoing extreme shearing and being cultured for up to five days, esophageal cancer cells maintained their differentiation state, and the tumor structural integrity was well preserved. Although there was a reduction in both the number and volume of tumor cells in comparison to the pre - HDRA, accompanied by a certain degree of necrosis. The proliferation of tumor tissue was evaluated through Ki-67 immunostaining, revealing that the proliferative capacity remained relatively stable both before and after HDRA procedure, with no significant decrease noted. Moreover, the expression of CD31 and E-cadherin demonstrated that the interstitial structure, microenvironment, differentiation, and invasion characteristics of the tumor tissues remained consistent with those of the original tumor tissue. The HDRA experiment demonstrated strong feasibility and stability, making it suitable for routine clinical applications to guide patients in individualized treatment.

### Efficacy rate for anticancer agents measured by HDRA

A total of 283 gastrointestinal tumor patients were included in this study, comprising 124 cases of esophageal cancer, 92 cases of cardia cancer/gastric cancer, and 67 cases of colorectal cancer. The experimental drug regimens and their corresponding efficacy rates, calculated as the number of chemosensitive cases divided by the number of evaluated cases, are presented in **Table 1**. For esophageal cancer, cardia/gastric cancer, and colorectal cancer, the efficacy of combination therapies was significantly higher than that of monotherapies. The efficacy rates of combination therapies ranged from 30% to 50%. In fact, the efficacy of drug treatment following combination therapy increased by 30%. Furthermore, combinations of three or four drugs exhibited even greater efficacy, potentially reaching 80% to 90%. This indicated that combined medications were more likely to enhance patient sensitivity. However, not all drug combinations enhanced efficacy rate, e.g., LBP vs ALT combined with LBP. In esophageal cancer, drug regimens including platinum-based drugs (such as CDDP, CBP and OXA) showed higher efficacy rate. Among these, CDDP demonstrated particularly outstanding performance. When CDDP was combined with 5-FU/TS-1/XEL/TAX/DTAX, all of which demonstrate efficacy rates exceeding 60%. In cardiac/gastric cancer, several schemes related to TAX or OXA had a relatively high efficacy rate. In the treatment of colorectal cancer, drug combination regimens involving CPT-11 or OXA demonstrated relatively high efficacy rates. This indicated that the efficacy of appropriate drug combination therapy for gastrointestinal tumors was superior to that of monotherapy.

**Table 1:** Efficacy rate obtained by HDRA with MTT endpoint in gastrointestinal tumor.

Esophagus cancer treatment agent	Efficacy rate (HDRA with MTT endpoint)	
TAX	45/124	32.3%
ALT	28/110	25.5%
CBP	55/110	50.0%
LBP	67/110	60.9%
5-FU	7/14	50.0%
TAX+CBP	69/110	62.7%
TAX+LBP	68/110	61.8%
TAX+CDDP	78/110	70.9%
ALT+CBP	58/110	52.7%
ALT+LBP	63/110	57.3%
ALT+CDDP	53/110	48.2%
CDDP+5-FU	12/14	85.7%
CDDP+XEL	10/14	71.4%
CDDP+TS-1	11/14	78.6%
CDDP+DTAX	9/14	64.3%
5-FU+OXA	13/14	92.9%
5-FU+CPT-11	12/14	85.7%
5-FU+LEU+OXA+DTAX	13/14	92.9%
Cardiac/gastric cancer treatment agent	Efficacy rate (HDRA with MTT endpoint)	
TAX	37/93	39.8%
TS-1	29/51	56.9%
DTAX	33/51	64.7%
OXA+TS-1	39/51	76.5%
TAX+CBP	64/93	68.8%
TAX+LBP	34/42	81.0%
TAX+CDDP	23/42	54.8%
TAX+5-FU	39/51	76.5%
5-FU+CDDP	41/51	80.4%
5-FU+DTAX	31/51	60.8%
5-FU+OXA+LEU	42/51	82.4%
5-FU+DTAX+OXA+LEU	41/51	80.4%
Colorectal cancer treatment agent	Efficacy rate (HDRA with MTT endpoint)	
5-FU	37/67	55.2%
XEL	35/67	52.2%
OXA	34/67	50.7%
CPT-11	34/67	50.7%
XEL+OXA	43/67	64.2%
XEL+CPT-11	51/67	76.1%
OXA+5-FU+LEU	48/67	71.6%
CPT-11 + 5-FU+LEU	49/67	73.1%
CPT-11 + 5-FU+LEU+OXA	59/67	88.1%

### Inhibition rate of drugs measured by HDRA with MTT endpoint

**Figure 2:**
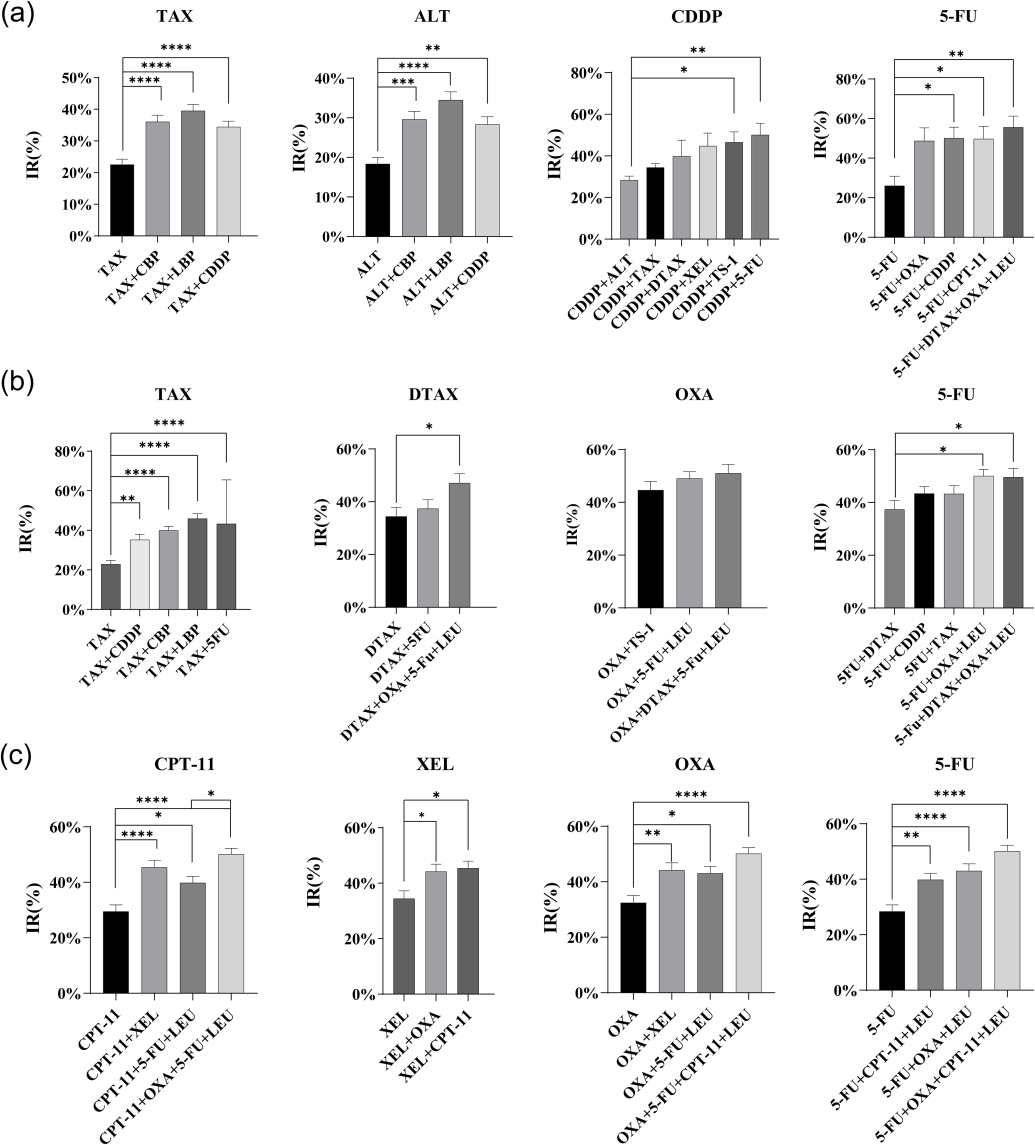
Comparison of inhibitory rates (IRs) of single and combination drug regimens. **(a)** Comparison of IR average values for each drug regimen in esophageal cancer. **(b)** Comparison of IR average values for each drug regimen in cardiac/gastric cancer. **(c)** Comparison of IR average values for each drug regimen in colorectal cancer.(ANOVA test or Kruskal-Wallis test,*p<0.05, ** p<0.01, *** p<0.001, **** p<0.0001).

Besides assessing the efficacy rates of treatment regimens, we also evaluated the inhibition rates of several classical drugs and their combinations in 124 patients with esophageal cancer, 92 patients with gastric cancer, and 67 patients with colorectal cancer. These inhibition rates were determined through the MTT assay by measuring the absorbance values. Among the commonly used clinical drugs for esophageal cancer treatment, we selected four drugs-TAX, ALT, CDDP, and 5-FU to analyze their inhibition rates in both monotherapy and combination therapies. The inhibition rate significantly increased when TAX and ALT were combined with platinum-based drugs, especially the combination with LBP demonstrating the most pronounced effect. In cisplatin-based combination treatment regimens, the pairing of CDDP and 5-FU achieved the highest inhibition rate, while the combination with ALT exhibited the lowest inhibition rate, with a statistically significant difference. What’s more, the inhibition rate of 5-FU in multi-drug combinations was significantly higher than that observed in single-drug or two-drug combinations (**Figure 2a**). In the treatment of cardiac/gastric cancer, the inhibition of TAX combination therapy was significantly higher than monotherapy. Among these, the combination of TAX and LBP exhibited the highest inhibition rate, which was consistent with the findings of TAX combination therapy in esophageal cancer. Additionally, multi-drug combinations of DTAX or 5-FU demonstrated better efficacy than monotherapy. However, they did not show a significant advantage over dual-drug combinations (**Figure 2b**). In the treatment of colorectal cancer, the inhibition rates of CPT-11/XEL/OXA/5-FU combination therapy were significantly higher than those of monotherapy. Notably, the inhibition rate of CPT-11 combined with OXA, 5-FU, and LEU was significantly greater than that of CPT-11 combined with 5-FU and LEU, suggesting that the addition of OXA enhances the synergistic effect. However, in OXA-based regimens, the efficacy of OXA combined with CPT-11, 5-FU, and LEU was not significantly superior to that of OXA combined with 5-FU and LEU (**Figure 2c**). These findings indicated that the inhibition rate of appropriate drug combination therapy for gastrointestinal tumors was superior to that of monotherapy.

### Inhibition rates in subgroup analyses stratified by T, N, TNM stages and Ki-67 expression

**Figure 3:**
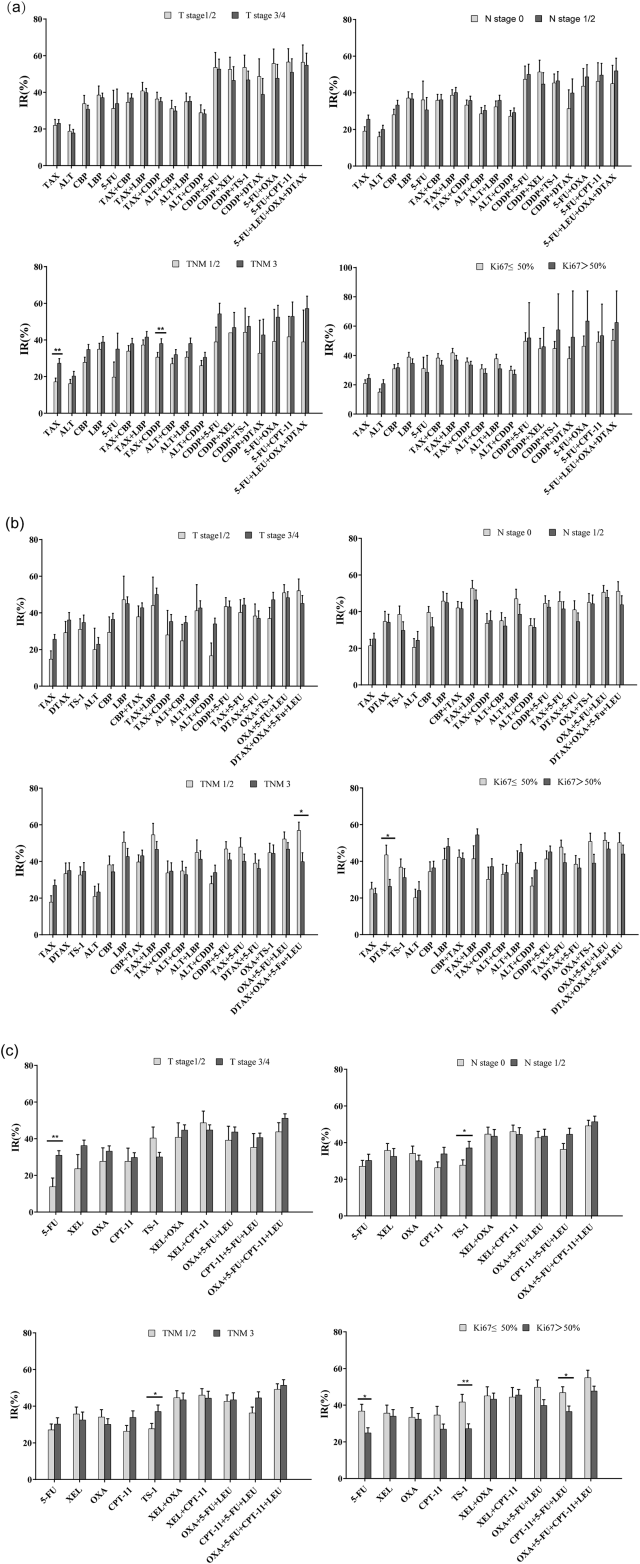
Comparison of inhibitory rates (IRs) according to primary tumor (T), regional lymph nodes (N), TNM staging, and Ki-67 index. **(a)** Subgroup analysis of esophageal cancer based on T, N, TNM, Ki-67; **(b)** Subgroup analysis of cardia/gastric cancer based on T, N, TNM, Ki-67; **(c)** Subgroup analysis of colorectal cancer based on T, N, TNM, Ki-67 (Independent Samples t - test or Kruskal-Wallis test, *p<0.05, **p<0.01).

According to the AJCC tumor staging standards, patients were categorized based on the primary tumor (T), regional lymph nodes (N), TNM staging, and Ki-67 index. The inhibition rates of the same regimen in different subgroups were then compared. In the TNM subgroups of esophageal cancer, the IR of the drugs tends to be high relatively higher as the TNM stage advanced. Notably, the IR of the TAX and TAX combined CDDP regimen was significantly higher in patients with stage III cancer compared to those with stage I or II (**Figure 3a**). In the subgroup analysis of cardia/gastric cancer, the IR of DTAX combined with OXA, 5-FU, and LEU was higher in the TNM I/II stages, and DTAX exhibited a higher IR in the group with Ki-67 ≤ 50% (**Figure 3b**). In the subgroup analysis of colorectal cancer, the IR of 5-FU was significantly higher in T stage 3 and 4 compared to T stage 1 and 2. Moreover, the IR of TS-1demonstrated a positive correlation with both N and TNM stages, while exhibiting a negative correlation with Ki-67 expression level. Similarly, the IR of 5-FU and CPT-11 combined with 5-FU and LEU decreased as the expression level of Ki-67 increased (**Figure 3c**). Additionally, subgroup analyses were performed based on gender. The IR of DTAX combined with 5-FU was higher in female patients with cardia/gastric cancer. Conversely, the IR of OXA and XEL combined with CPT-11 increased significantly in male patients with colorectal cancer (**Supplementary Figure 1**). Therefore, tumor staging, proliferative activity, and gender significantly influenced patients’ inhibition rates (IR) and drug sensitivity to specific therapeutic agents.

### Survival and prognosis for patients in HDRA - guided treatment

According to the actual clinical treatment, patients were categorized into three groups: the HDRA group, the N-HDRA group, and the untreated group. A retrospective analysis was conducted to assess the prognosis of patients across these three groups. The characteristics of the patients in each group are presented in **Table 2**. No significant differences were observed among the three groups in terms of gender, age, stage, vascular invasion, neural invasion, p53, and Ki-67, whether the cancer type was esophageal cancer, cardia/gastric cancer, or colorectal cancer. Furthermore, in the observational indicators for gastric and colorectal cancers, HER-2 overexpression and microsatellite instability (MSI) were assessed, and no significant inter-group differences were noted.

**Table 2:** The characteristics of patients with gastrointestinal cancer grouped by treatment.

Characteristics	HDRA Group	N-HDRA Group	Untreated Group	*P* value
Esophagus cancer	31	32	61	
Males/females	24/7	29/3	50/11	0.358
Age (mean ± SD)	66.00 ± 8.93	67.80 ± 7.40	69.56 ± 6.64	0.133
pStageIIA/IIB/IIIA/IIIB/IIIC	8/3/6/4/10	9/6/9/4/4	25/7/16/11/3	0.074
Vascular infiltration	24	19	40	0.299
Nerve infiltration	15	13	33	0.464
P53 (%)	75.00 ± 22.10	60.00 ± 23.71	70.00 ± 28.77	0.482
Ki-67 (%)	60.00 ± 18.32	55 ± 20.20	52.31 ± 21.27	0.194
Cardiac/gastric cancer	25	38	29	
Males/females	21/4	33/5	26/3	0.827
Age (mean ± SD)	64.50 ± 9.43	68.00 ± 10.56	69.00 ± 7.99	0.094
pStageIIA/IIB/IIIA/IIIB/IIIC	5/6/1/7/6	8/5/7/10/8	5/6/2/9/7	0.756
Vascular infiltration	16	30	19	0.339
Nerve infiltration	16	27	19	0.815
Her-2 overexpression	1	5	1	0.243
MSI	10	15	16	0.381
P53 (%)	20.00 ± 37.71	50.00 ± 35.22	65.00 ± 37.07	0.533
Ki-67 (%)	50.00 ± 18.67	60.00 ± 17.50	60.00 ± 16.82	0.128
Colorectal cancer	28	21	18	
Males/females	22/6	10/11	11/7	0.078
Age (mean ± SD)	60.64 ± 12.27	64.10 ± 10.38	66.67 ± 14.26	0.261
pStageIIA/IIB/IIIA/IIIB/IIIC	12/3/1/5/7	7/2/2/3/6	11/2/0/3/0	0.134
Vascular infiltration	18	9	6	0.095
Nerve infiltration	8	3	5	0.436
Her-2 overexpression	9	5	2	0.236
MSI	4	2	1	0.617
P53 (%)	20.00 ± 38.15	30.00 ± 35.01	57.5 ± 37.30	0.349
Ki-67 (%)	65.00 ± 17.40	60.00 ± 19.15	75.00 ± 14.62	0.109

**Figure 4:**
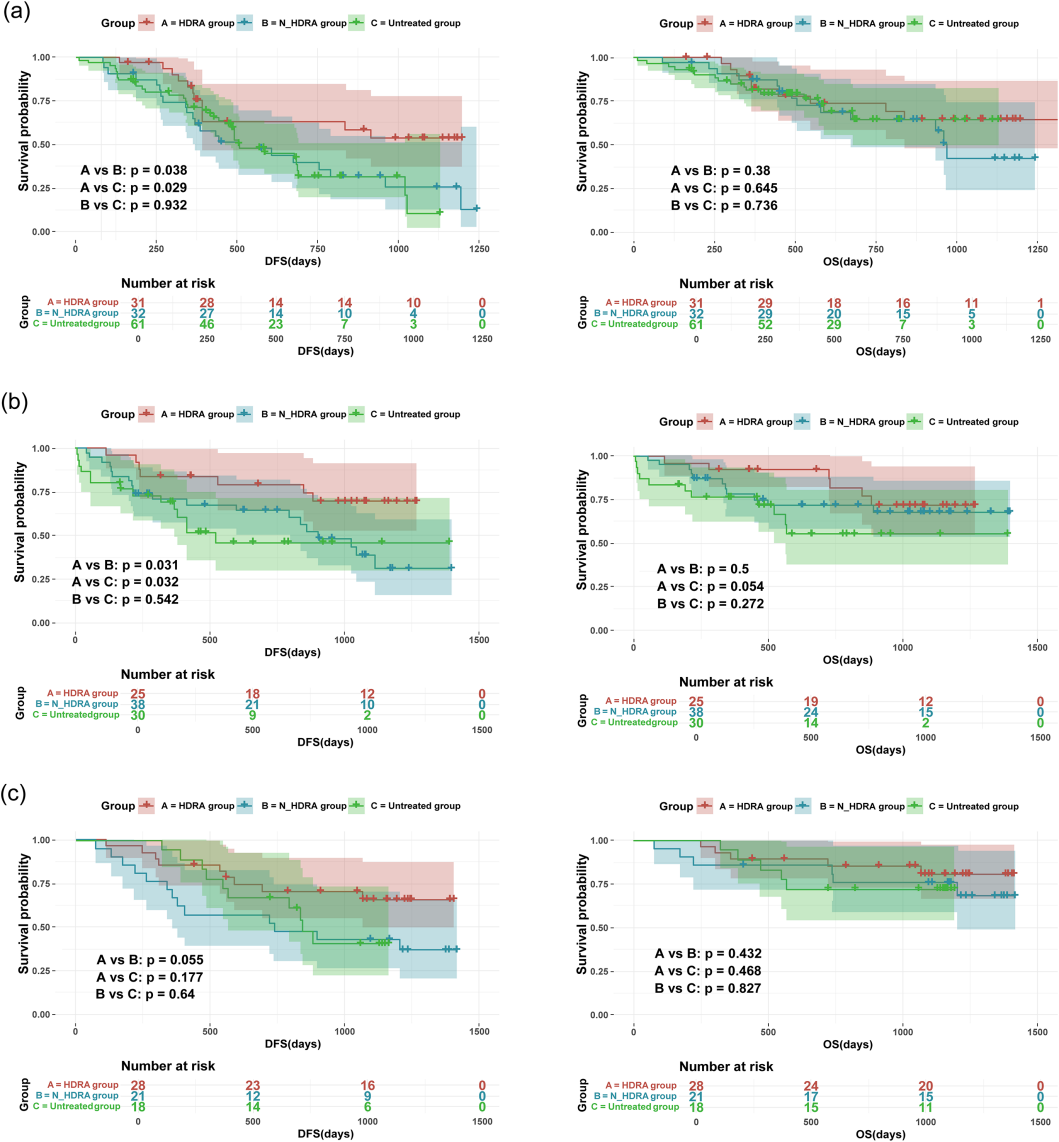
Kaplan–Meier survival curves showing disease-free survival (DFS) and overall survival (OS) of patients receiving the HDRA-sensitive regimen(HDRA), the non-HDRA-sensitive regimen or no treatment. **(a)** the survival analysis of esophageal cancer; **(b)** the survival analysis of cardiac/gastric cancer; **(c)** the survival analysis of colorectal cancer.

The Kaplan-Meier method was employed to analyze disease-free survival (DFS) and overall survival (OS) in patients (**Figure 4**). In patients with esophageal cancer, the DFS of the HDRA group (846 days) was significantly higher than that of both the N-HDRA group (638 days, p=0.038) and the untreated group (608 days, p=0.029). However, no statistically significant difference in DFS was observed between the N-HDRA group and the untreated group (p=0.932). Similarly, in the survival analysis of cardia/gastric cancer, the DFS in the HDRA group (1023 days) was significantly higher than that in both the N-HDRA group (832 days, p=0.031) and the untreated group (769 days, p=0.032), with the difference being statistically significant. However, there was no significant difference in DFS between the N-HDRA group and the untreated group (p=0.542). In colorectal cancer, we found that the DFS in the HDRA group (1099 days) exceeded that of both the N-HDRA group (818 days, p=0.055) and the untreated group (845 days, p=0.177). However, no statistically significant differences were noted among these groups. These findings indicated that the DFS of patients treated with the HDRA sensitive regimen was significantly superior to that of patients receiving not sensitive therapy or no treatment. We also observed the median overall survival (OS) of the patients. By the end of the follow-up period, OS for patients with esophageal cancer, gastric cancer, and colorectal cancer in the HDRA group was 1035 days, 1086 days, and 1250 days, respectively. These values were all higher than those in the N-HDRA group, which had 885 days for esophageal cancer, 1062 days for gastric cancer, and 1125 days for colorectal cancer, as well as the untreated group, with 860 days for esophageal cancer, 895 days for gastric cancer, and 988 days for colorectal cancer. However, there were no statistically significant differences between the groups (p > 0.05).

**Figure 5:**
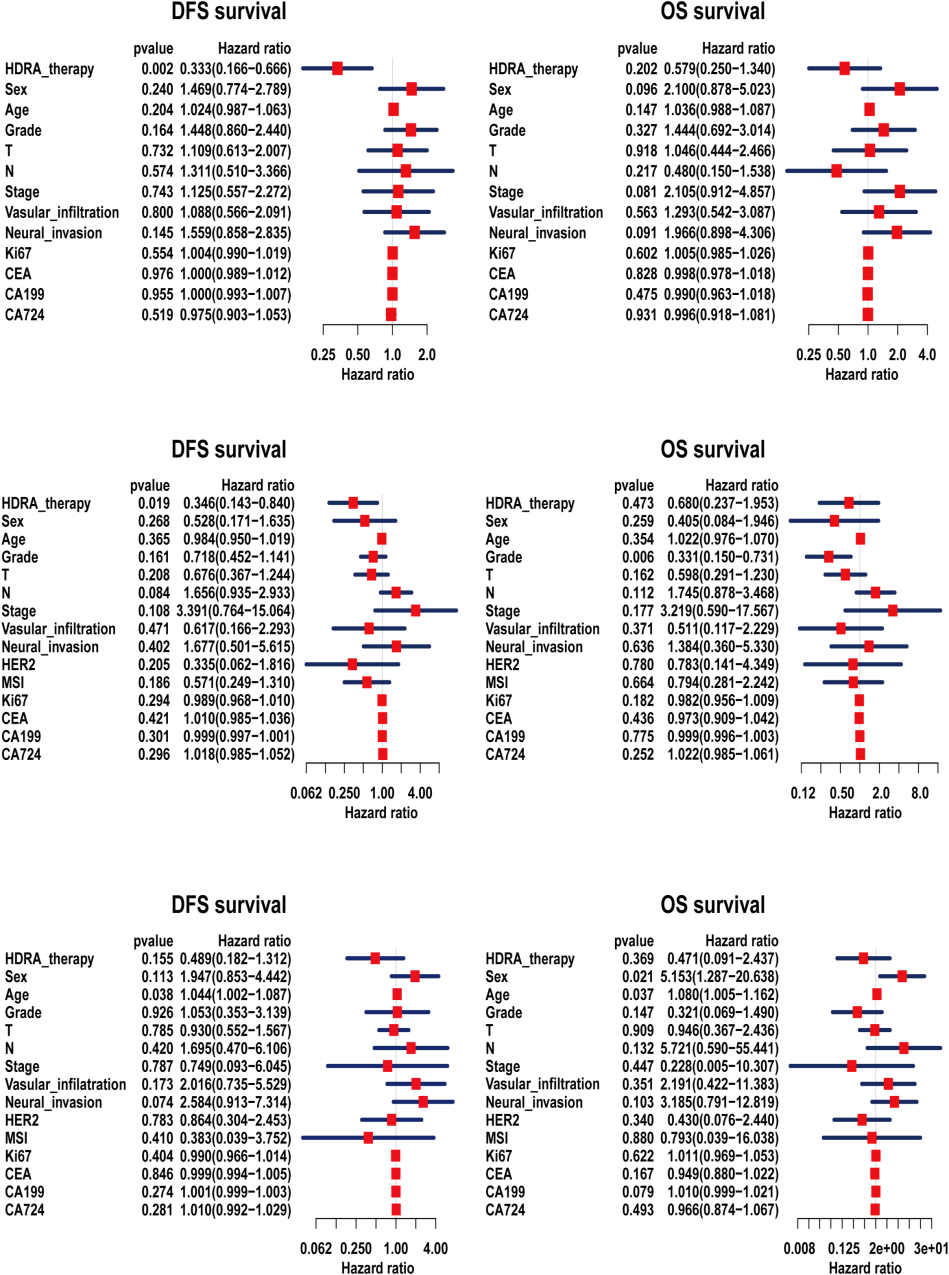
Cox regression analysis of disease-free survival (DFS) and overall survival (OS) for patients based on HDRA-sensitive therapy. **(a)** the cox analysis of esophageal cancer; **(b)** the cox analysis of cardiac/gastric cancer; **(c)** the cox analysis of colorectal cancer.

We also conducted Cox regression analysis (**Figure 5**). The result revealed that treatment guided by the HDRA-sensitive regimen was a protective factor for patient survival (Hazard ratio, HR < 1). Furthermore, in the regression models of DFS for patients with esophageal and cardia/gastric cancers, the influence of HDRA-guided clinical treatment demonstrated significant statistical differences. In the analysis of OS, HDRA-guided therapy was found to reduce the risk of mortality events. Nevertheless, this reduction did not reach statistical significance.

## Discussion

Tumor progression is critically dependent on the tumor microenvironment (TME) (25). This complex milieu exerts profound and multifaceted influences on therapeutic responses through various mechanisms, including the extracellular matrix (ECM), which acts as a physical barrier to drug diffusion; aberrant vascular networks that compromise drug delivery efficiency; and metabolic symbiosis that promotes drug resistance (26, 27). Consequently, preserving the integrity of the TME in ex vivo culture systems is essential for restoring the biological characteristics of tumors, significantly enhancing the predictive accuracy of drug screening and mitigating the risk of false positives or negatives that may arise from the absence of microenvironment (28). The immunohistochemical results demonstrated that the histoculture drug response assay (HDRA) with MTT endpoint effectively preserves the three-dimensional architecture and tumor microenvironment of gastintestinal cancer tissues. Furthermore, it conforms that HDRA is a reliable method for evaluating accurate chemotherapy regimens, thereby providing guide for individual treatment.

The HDRA results indicated that across all gastrointestinal tumor subtypes, the efficacy and inhibition rates of combination chemotherapy regimens were significantly superior than those of monotherapy, which aligns with findings from other studies (23). However, not all combinations enhance the drug inhibition rate. In esophageal cancer, the combination of platinum with paclitaxel or 5-FU demonstrates notable efficacy, consistent with the drugs recommended in clinical guidelines (29). Nevertheless, this treatment regimen exhibits varying inhibition rates among different patients. Therefore, it is essential to conduct HDRA to identify the most suitable combined treatment regimen for individual patients rather than relying on arbitrary combinations. Similarly, it is inappropriate to select combined treatment regimens solely based on clinical experience in the management of gastric cancer and colorectal cancer. The HDRA-guided treatment selection can effectively avoid ineffective treatment strategies and genuinely facilitate individualized therapy.

This study extensively explores the impact of clinical characteristics on the responsiveness to chemotherapy drugs, revealing the complexity and individual differences in the treatment of gastrointestinal cancer. Subgroup analyses indicate that TNM stage, Ki-67 expression levels, and gender influence the inhibition rate of specific regimens. This may be due to tumor progression or target genes alterations affect the capacity of drugs to penetrate tumors (30–32). Consequently, it is essential to consider the clinical characteristics of patients when formulating treatment strategies. HDRA assay integrates specific tumor characteristics and drugs sensitivity of patients across multiple aspects to offer more precise and effective treatment plans (33, 34). Patients treated under the guidance of HDRA exhibited a significantly prolonged disease-free survival (DFS). Furthermore, COX regression analysis confirmed that HDRA treatment serves as a protective factor for survival. The lack of significance in overall survival (OS) may be attributed to a limited follow-up duration or confounding factors, such as subsequent therapies post-recurrence. Clinically, our findings advocate for the integration of HDRA with MTT endpoint into routine practice to minimize the reliance on empirical chemotherapy. By identifying non-responders at an early stage, HDRA has the potential to reduce unnecessary toxicity and financial burdens, particularly for regimens with low efficacy and inhibition rates. Furthermore, the assay’s capability to evaluate drug combinations provides a valuable platform for testing novel therapeutic strategies, such as the combination of targeted agents with traditional cytotoxic drugs.

While this study provides compelling evidence for the clinical utility of HDRA, several limitations need to be attention. First, this study exclusively involved Chinese patients, which may limit the generalizability of the findings to other ethnic or geographic populations. Second, due to institution-specific clinical practice patterns, certain therapeutic regimen choices were subject to inherent bias and may not fully align with guideline-recommended treatments. Third, the sample sizes for specific quadruple-drug combinations were relatively small, highlighting the need for future multi-center prospective studies with standardized treatment protocols. Additionally, the exclusion of stage IV patients restricts the applicability of the results to advanced metastatic disease. Furthermore, the follow-up period (median 22.87 months) may underestimate long-term survival differences. The conventional HDRA assay has certain limitations, as it fails to fully simulate the dynamic host-tumor interactions between host and tumor. Exploring the integration of nanotechnology with 3D tissue models based on HDRA could significantly enhance the predictive capability of chemotherapy through precise drug delivery, microenvironment simulation, and the integration of diagnostics and treatment (35–37). Additionally, conducting correlational analysis with genomic biomarkers, such as PD-L1 and MSI, may improve predictive accuracy, thereby providing valuable guidance for personalized cancer treatment.

## Conclusion

HDRA is a reliable tool for predicting chemosensitivity and tailoring individualized therapies for gastrointestinal cancers. By preserving tumor biology and identifying synergistic drug combinations, HDRA-guided regimens enhance disease-free survival (DFS) and minimize ineffective treatments. Future efforts should concentrate on expanding clinical validation, refining assay protocols, and exploring the integration of HDRA with emerging therapeutic modalities such as immunotherapy. These advancements will further solidify HDRA in precision therapy for gastrointestinal cancers.

## Data Availability

The raw data supporting the conclusions of this article will be made available by the authors, without undue reservation.
