# Supplementary material for: Histoculture drug response assay predicts chemotherapy efficacy and improves survival in gastrointestinal cancers
Source: Front Oncol. 2025 Jul 16;15:1596253. doi: 10.3389/fonc.2025.1596253 (PMC12307191; doi:10.3389/fonc.2025.1596253)
Supplement: Supplementary file 1 [file DataSheet1.docx]

Supplementary Material


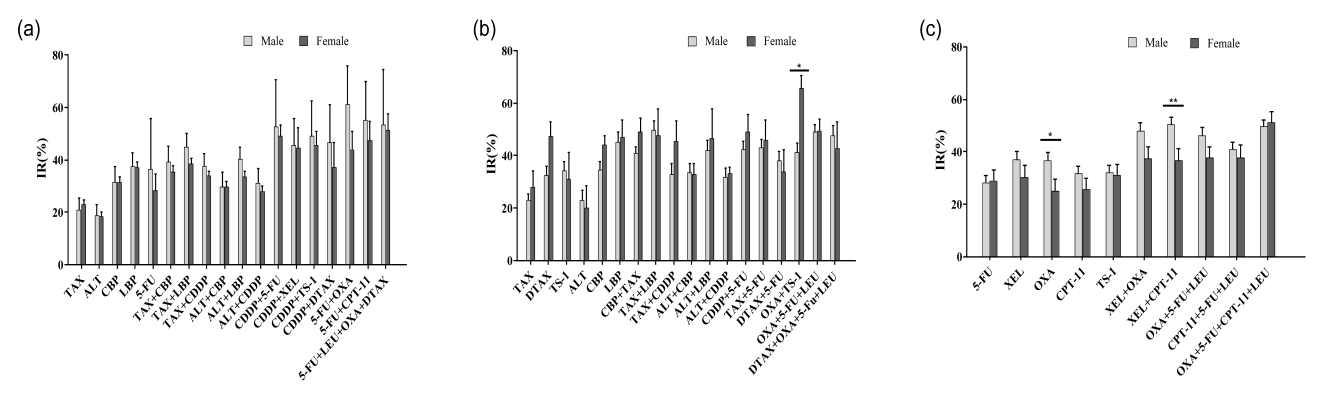


**Supplementary Figure 1.** Comparison of inhibitory rates (IRs) based on gender.(a)Subgroup analysis of esophageal cancer.(b)Subgroup analysis of cardia/gastric cancer .(c)Subgroup analysis of colorectal cancer.(Independent Samples t - test or Kruskal-Wallis test, *p<0.05, ** p<0.01).
